# Disorganized hippocampal excitatory and inhibitory connectivity in a mouse model of Alzheimer’s disease

**DOI:** 10.1016/j.ibneur.2025.09.004

**Published:** 2025-09-19

**Authors:** Tetsufumi Ito, Munenori Ono, Sachiko Yamaki, Yoshie Hori, Shinji Muramoto, Ryo Yamamoto, Takafumi Furuyama, Nobuo Kato

**Affiliations:** a Department of Systems Function and Morphology, University of Toyama, Toyama 930-0194, Japan; b Department of Physiology, Kanazawa Medical University, Uchinada 920-0293, Japan

**Keywords:** Alzheimer’s disease, Electron microscopy, Spatial learning, Hippocampus, Neuronal excitability

## Abstract

Cortical hyperexcitability is regarded to accompany Alzheimer’s disease (AD) and its rodent models, and often claimed to be even causative of AD. To seek its morphological backgrounds, spatial learning was assessed with the Morris water maze test (MWM) in male 3xTg Alzheimer’s model mice of 5–6 months old, then their hippocampal tissue was examined by electron microscopy (EM). By assigning EM-based asymmetric and symmetric synapses to excitatory (E) and inhibitory (I) synapses, respectively, and by attributing synapses on spines to those onto excitatory (E) postsynaptic neurons, we defined 2 different types of synapses: E-to-E and I-to-E synapses. In addition, synapses onto non-spinous structures (N) of postsynaptic neurons were designated as E-to-N or I-to-N. We thus categorized hippocampal synapses into 4 classes. I-to-E synapses were 7-fold denser in 3xTg than in wild-type mice, whereas the other types did not differ in density. In MWM, there was a non-significant tendency that AD mice perform worse than WT mice. We found a non-significant tendency for the E-to-E synapse density to correlate inversely with MWM performance in AD mice, though the correlation was significant with AD and WT mice pooled together. When E-to-E and E-to-N synapses are combined as the asymmetric synapse class, the density was significantly correlated in AD mice isolated. The I-to-E synapse density in AD mice exhibited the tendency to inverse correlation with MWM performance. Overall, categorizing hippocampal synapses into 4 classes, we confirmed from a new angle the received view that a higher hippocampal excitability could deteriorate cognition.

## Highlights

• Cortical hyperexcitability accompanies Alzheimer’s disease (AD) brains.
• Hippocampal synapses were examined and counted with electron microscopy.
• Density of excitatory synapses on spines was inversely correlated with memory.
• Excessive spinous excitatory synapses may raise excitability and impair memory.
• Inhibitory synapses on spines increase in AD mice.

## Introduction

In familial Alzheimer’s disease (AD), pathogenic mechanisms appear to be well understood based on responsible mutations on key enzymes and substrates that contribute to AD pathogenesis. According to the amyloid hypothesis (Hardy and Selkoe, 2002, Tomiyama et al., 2008, Perrin et al., 2009, Karran et al., 2011, Jack, 2022), AD pathogenesis starts with Aβ overproduction. Then, soluble oligomeric Aβ gradually urges tau synthesis and its phosphorylation, eventually deteriorating neuron circuits. For familial AD, genetics mostly determine how the initial Aβ production is started (Benilova et al., 2012). For sporadic AD, by contrast, the initiation process remains elusive. One of the candidates that may trigger the initial Aβ production is an increase in neuronal excitability. Aβ production is known to depend on synaptic activity (Cirrito et al., 2005). Aβ in turn is demonstrated to increase neuronal excitability (Yamamoto et al., 2011, Scala et al., 2015). Thus, a vicious cycle emerges, in which activity-dependent Aβ production elevates neuronal excitability, thereby accelerating Aβ production further. Indeed, cortical hyperexcitability and epileptogenesis have been documented in AD patients (Perretti et al., 1996, Ferreri et al., 2003, Inghilleri et al., 2006), as well as in mouse models (Palop et al., 2007, Busche et al., 2008, Minkeviciene et al., 2009; Born, 2015).

It has been well established that soluble Aβ leads to neuronal toxicities, including synaptic inhibition (Hsia et al., 1999, Kamenetz et al., 2003), abolished plasticity (Walsh et al., 2002), glutamate receptor decrease (Snyder et al., 2005, Hsieh et al., 2006), and synapse loss (Kuo et al., 1996, Lambert et al., 1998). In many studies on various kinds of AD mice, spine loss is often reported and regarded as the surrogate for synapse loss (Wei et al., 2010, Mairet-Coello et al., 2013, Herzer et al., 2018, Wang et al., 2023), although spines represent synapses on excitatory neurons only. Synapses on non-spine structures or inhibitory asymmetric synapses were investigated only in a limited number of EM studies (Keller et al., 2025). The present experiments used electron microscopy (EM) to characterize hippocampal synapses in 3xTg-AD model mice from two points of view: (1) asymmetric or symmetric synapses, which represent excitatory and inhibitory (E and I) synapses; (2) spinous or non-spinous synapses (N). We considered that spinous synapses terminate onto postsynaptic excitatory neurons (E) based on the received view that most spines except only few are carried by excitatory neurons in the neocortex (Petilla Interneuron Nomenclature Group, 2008; Rudy et al., 2011; Tremblay et al., 2016) and hippocampus (Freund and Buzsáki, 1996, Gulyás et al., 1999, Scheuss and Bonhoeffer, 2014). In this way, we singled out 2 types of spinous synapses onto excitatory postsynaptic neurons (E-to-E and I-to-E). The rest correspond to non-spinous synapses, which were provisionally designated to E-to-N and I-to-N synapses depending on asymmetric or symmetric EM images, although the postsynaptic nature of these synapses was left undetermined. We then examined correlation between the density of each synapse type and spatial learning in AD mice. However, only with E-to-E and E-to-N synapses combined as the asymmetric synapse class, we were able to find a significant correlation in AD mice. The rare type of I-to-E synapses on spines turned out to be 7-fold denser in AD than wild-type mice.

## Materials and methods

### Animals

All the experiments were performed in accordance with the guiding principle of the Physiological Society of Japan and with the approval of the Animal Care Committee of Kanazawa Medical University. Triple transgenic AD model mice (3xTg) with 129/C57BL6 hybrid background (Oddo et al., 2003), provided from Dr LaFerla (University of California, Irvine) *via* Dr Ohyagi (Ehime University, Japan), were kept in group cages in our in-house colony under a 12:12 h day-night cycle, and given free access to food and water. We used male 3xTg mice of 5–6 months of age. As the age-matched control, non-transgenic mice from the same hybrid background were used (wild-type; WT).

### Behavioral tests

The Morris water maze test was performed by using a round tank (120 cmφ) filled with opaque water (25°C). A plastic platform (10 cmφ) was placed below the water surface. Several landmarks including 4 large, differently-colored objects were placed around the tank. For the escape training, mice were given 4 sessions of swimming on each of 5 consecutive days. For each session, the mice were released from a starting point pseudo-randomly chosen from the 4 prefixed positions on the edge of the tank separated 90° from one another, and the time spent to reach the platform was measured as the escape latency. Unless mice reached the platform within 60 s by themselves, they were placed on the platform by the experimenter for 15 s. The average time spent over the 4 sessions yielded the latency score on a particular day for an individual mouse. The day-by-day averages were then calculated for each group. The progress of the learning for each animal was analyzed by an exponential curve fitting with Origin software (Origin 2019, OriginLab Corporation, Northampton, MA), yielding the individual learning slope under the fit function Exp2pmod2. The probe test was performed, with the platform removed, subsequently to the last escape session on the 5th day. The animals were placed into the water from the edge of the pool located farthest from the former platform position and allowed to swim for 1 min. The swimming trajectory was videotaped and analyzed offline by SMART software (SMART, Panlab s.l.u., Cornella, Spain). The time spent in the target quadrant was calculated and expressed as percentage over the total duration.

### Electron microscopy

After the behavioral test, mice were deeply anesthetized by sodium pentobarbital (150 mg/1 kg, i.m.), and perfused transcardially with 4 % paraformaldehyde in PBS (pH 7.4) and then with a 30 % sucrose solution. Brains were removed, stored at 4°C for 2–3 weeks, and cut at 20 μm with a freezing microtome. The specimen was handed at this stage to those who did EM analysis without notification on details of the behavioral results. For EM sectioning, the sections were postfixed for 30 min with 1 % osmium tetraoxide diluted in 0.1 M phosphate buffer (pH 7.4), and stained *en bloc* with uranyl acetate overnight. Then the sections were dehydrated with graded ethanol, substituted with propylene oxide, and embedded in Epok812 (Oken Shoji, Japan). The Hippocampal CA1 region was trimmed, and serial ultrathin sections were made with an ultramicrotome (EM FCS, Leica Microsystems, Germany) and observed with an electron microscope (EM; H7650, Hitachi, Japan).

### EM analysis

We set 100 μm × 100 μm grids on an ultrathin section for each animal, and an electron micrograph (image size: 3.01 μm × 3.01 μm) was acquired in the center of each grid at × 6000 magnification. The number of electron micrographs for each animal ranged from 20 to 87 (average: 48.9; N = 10). We identified asymmetric synapses by thin postsynaptic density and the presence of pleomorphic vesicles in presynaptic sites. Likewise, we identified symmetric synapses by the thick postsynaptic density and presence of small round vesicles in presynaptic sites. We categorized the postsynaptic regions into 3 classes, namely spine, dendritic shaft/soma, and unidentified. We identified soma as the dense distribution of rough endoplasmic reticulum, and dendritic shaft as the presence of neurofilaments and microtubules. Dendritic spines were identified as the absence of neurofilaments or microtubules. We calculated the density of synapses by dividing the mean counts of synapses with the area of a micrograph (9.07 μm^2^) and then dividing by the section thickness to yield the value per cubic μm.

### Data statistics

Data were expressed as mean ± standard error of the mean (SEM). Two-sample *t*-test and repeated measures ANOVA were used for group-wise comparison (SPSS v21, IBM Japan, Tokyo). Origin 2019 and SPSS were used to obtain relevant parameters such as the correlation coefficient based on exponential curve fitting and correlation analysis. With a p-value less than 0.05, the difference was considered significant.

## Results

### Classification of four different synapse types based on EM

**Fig. 1:**
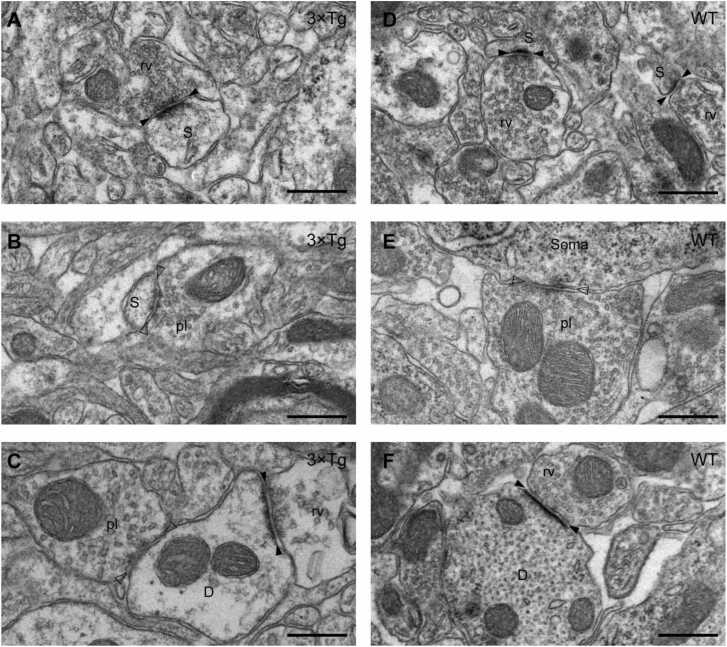
Electron micrographs of CA1 from 3 ×Tg (**A-C**) and wild-type (WT; **D-F**) mice. (**A**, **D**) Asymmetric axo-spinous synapse, which is characterized by thick postsynaptic density (black arrowheads), round vesicles (rv) in the presynaptic terminal, and the postsynaptic spine (S). (**B**) Symmetric axo-spinous synapse, which is characterized by similar pre- and postsynaptic densities (white arrowheads), pleomorphic vesicles (pl) in the presynaptic terminal, and the postsynaptic spine (S). Note that symmetric axo-spinous synapses were mainly found in 3 ×Tg, and very rare in WT mice. (**C**) Asymmetric and symmetric synapses on a dendritic shaft (D), which is identified by several microtubules. (**E**) Symmetric axo-somatic synapse. Note the presence of rough endoplasmic reticulum in postsynaptic cytosol (Soma). (**F**) Asymmetric synapse on a dendritic shaft, which is characterized by the presence of microtubules. Scale bars: 500 nm.

The ultrastructure of the CA1 region of the two genotype groups (Fig. 1) was examined after the behavioral experiments, but is described first for simplicity’s sake. We identified asymmetric and symmetric synapses by the shape of the synaptic cleft, the presence/absence of the thick postsynaptic density, and the type of synaptic vesicles in the terminals. We considered asymmetric and symmetric synapses to represent excitatory and inhibitory synapses. Furthermore, we identified postsynaptic structure by the presence/absence of microtubules, the putative landmark of the dendritic shaft, and the presence/absence of rough endoplasmic reticulum, the putative landmark of the spine.

We surveyed EM images and counted synapse numbers. Asymmetric synapses were, in general, most commonly formed on spines (Fig. 1A, D), which we thought conforms to excitatory-to-excitatory neuron (E-to-E) synapses. The density of those E-to-E synapses on spines was 2.162 ± 0.438/μm^3^ in WT (N = 4) and 2.417 ± 0.477/μm^3^ in AD mice (N = 6), clearly with no statistical difference. Asymmetric synapses on non-spinous (N) structures like dendrites were not rare (Fig. 1C, F), which we designated provisionally as E-to-N synapses (WT, 0.470 ± 0.050/μm^3^, N = 4; AD, 0.422 ± 0.085/μm^3^, N = 6). Symmetric synapses, which signal inhibitory synapses (I), ended mainly at non-spinous (N) structures including dendritic shafts (Fig. 1C) and somata (Fig. 1E), representing I-to-N synapses (WT, 0.240 ± 0.071/μm^3^, N = 4; AD, 0.274 ± 0.037/μm^3^, N = 6). By contrast, symmetric synapses on spines (Fig. 1B), which we designated to I-to-E synapses, were much less frequent than the other 3 types by an order of a tenth in WT mice (0.023 ± 0.001 /μm^3^, N = 4), but showed a 7-fold increase in AD mice (3xTg, 0.179 ± 0.016 /μm^3^, N = 6).

**Fig. 2:**
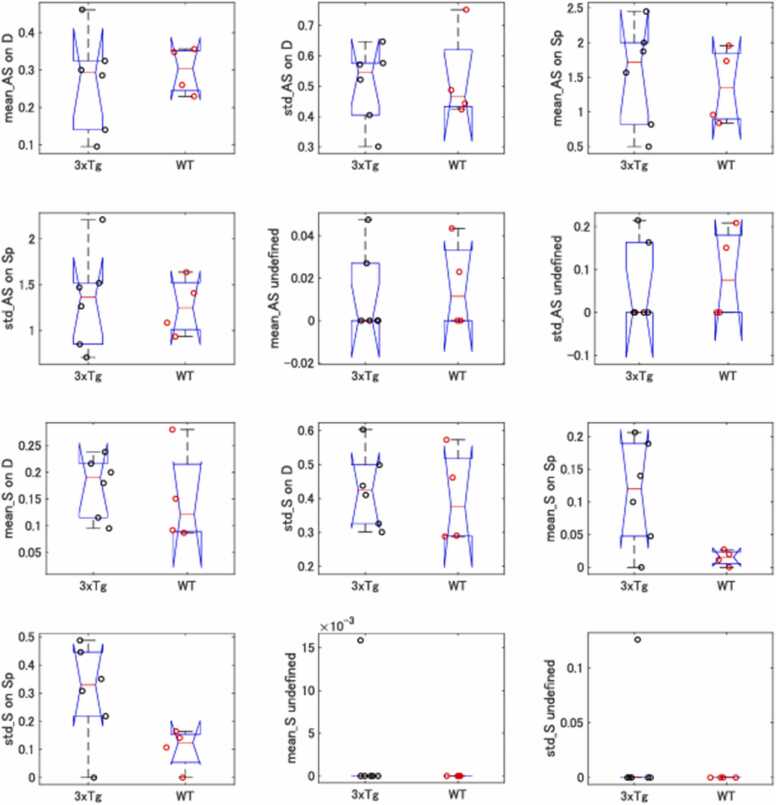
Notched box-whisker plot of the synaptic density. The average density (ordinate; synapse numbers/μm^3^) of each of the 4 different types of synapses (mean) is plotted and compared between 3xTg and WT groups. Its standard deviation (std) is platted in the following diagram. The density of synapses that cannot be identified is also plotted (Unidentified). Individual data points were illustrated with black (3xTg) and red (WT) open circles. The abbreviations ASOnD, ASOnSp, SOnD, and SOnSp signify asymmetric non-spinous synapses on dendrites and somata (E-to-N synapses), asymmetric synapses on spines (E-to-E), symmetric non-spinous synapses on dendrites and somata (I-to-N), and symmetric synapses on spines (I-to-E), respectively. The horizontal red bars indicate the medians. AS, asymmetric; s, symmetric; D, dendrites (also somata); Sp, spines.

The synapse density was compared between the WT and AD groups separately for the 4 different synapse groups: E-to-E, I-to-E, E-to-N, and I-to-N synapses. The mean density in individual mice and that of standard deviation were illustrated in the Notched box-whisker plot (Fig. 2), so as to enable visual comparison between the 95 % confidence margins of the two genotype groups. Our brief visual survey suggested that a statistical difference between the two genotype groups could exist only in the symmetric synapse on spine (Fig. 2, sOnSp, 2nd bottom on the right, blue rectangle), but obviously not for the other synapse types. We therefore compared the group-wise average density of this type of synapse (sOnSp; I-to-E synapses on spines) based on animal-wise average values, which showed the aforementioned 7-fold increase in AD mice as compared to the WT group. The difference turned out to be significant (P = 0.0313, *t*-test).

#### Correlation of learning performance and synaptic densities

**Fig. 3:**
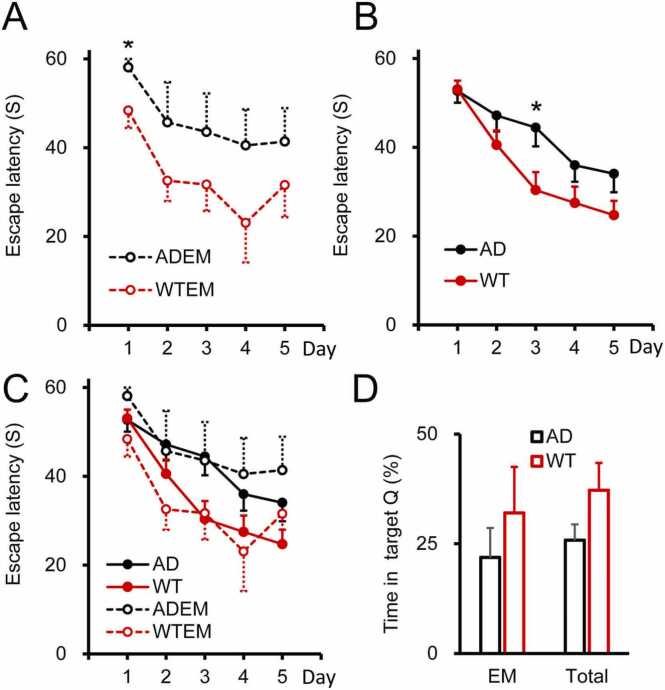
Morris water maze (MWM) performance. **A,** Day-by-day progress of MWM learning in AD (ADEM, black, N = 6) and WT mice (WTEM, red, N = 4) used for EM analysis. The asterisk on Day 1 indicates a significant difference (P < 0.05) in pairwise comparison after repeated measures ANOVA. **B,** MWM performance in extended numbers of AD (AD, black, N = 16 consisting of 6 EM mice with 10 newly added mice) and WT mice (WT, black, N = 14 consisting of 4 EM and 10 added mice). The asterisk on Day 3 indicates a significant difference (P < 0.05) as described in A. **C,** The diagrams in A and B are superimposed for comparison. The EM and extended groups follow basically the same day-by-day pattern, though variance is much larger in EM groups presumably because of the limited sample number. **D,** Probe session results. In both the EM and extended (Total) groups, WT mice had a non-significant tendency to perform better than AD mice.

In wild-type (N = 4) and 3xTg (N = 6) mice of 5–6 months old, we assessed spatial memory by using the Morris water maze (MWM) test. Between the two groups, we compared the daily shortening of the escape latency (Fig. 3A) and the time spent in the target quadrant in the probe trial (Fig. 3D). Repeated measures ANOVA revealed that there is no interaction across the genotype and daily progress (F=0.279, P = 0.890), though no difference was detected between the average values in the two groups (F=1.804, P = 0.216; Fig. 3A). Day-by-day comparison showed that there is a significant difference on Day 1 only (P = 0.038) but not on Days 2–5 (P > 0.05). The escape latency on the 5th day was 31.56 ± 7.19 s (WT, N = 4) vs. 41.38 ± 7.54 s (3xTg, N = 6, P = 0.3988, *t*-test). The relative time spent in the target quadrant during the probe session was 32.03 ± 10.49 % (WT, N = 4; WTEM in Fig. 3D) vs. 21.90 ± 6.74 % (3xTg, N = 6, P = 0.4162, *t*-test; ADEM in Fig. 3D). Although a tendency was clearly seen for 3xTg mice to be inferior in cognition to WT mice, the difference was not significant (Fig. 3A,D). This may be attributed to the limited number of mice, which were subjected to elaborate EM analysis as well. We therefore tested the MWM performance in a larger number of animals by newly adding WT (N = 10) and AD mice (N = 10) from the same age range (Fig. 3B,D). The finding confirmed the lack of significant difference in the escape latency on the 5th day between the two groups: 24.96 ± 3.17 s (WT, N = 14) vs. 34.05 ± 4.17 (3xTg, N = 16, P = 0.1011). In repeated measures ANOVA, there was no interaction across the genotypes and daily progress (F=2.024, P = 0.096), though a significant difference was narrowly undetectable between the average values in the two groups (F=3.999, P = 0.055; Fig. 3B). Day-by-day comparison showed that there is a significant difference on Day 3 only (P = 0.024) but not on the other Days (P > 0.05). Here again, although a tendency was seen, MWM performance did not significantly differ between the two groups (Fig. 3B,D). These results that we obtained from male only 3xTg mice appear to agree with a recent article from the founding lab of the 3xTg strain (Javonillo et al., 2022), which has reported that hippocampal long-term potentiation (LTP), a cellular model of learning, in slices obtained from male 3xTg mice up to 12 months old was impaired only marginally or not at all. Given such a well-preserved inducibility of LTP, the performance in male 3xTg mice of 5–6 months old, statistically not worse than the WT, would not be a peculiar phenomenon. Thus, not only did we examine the correlation between the cognitive performance and the density of each synapse type with the two genotypes of animals treated separately as should be (N = 6 vs. 4), but also with the two groups pooled together as a practical measure to draw a phenomenal picture from these statistically inseparable groups based on a larger number of EM samples (N = 10).

**Fig. 4:**
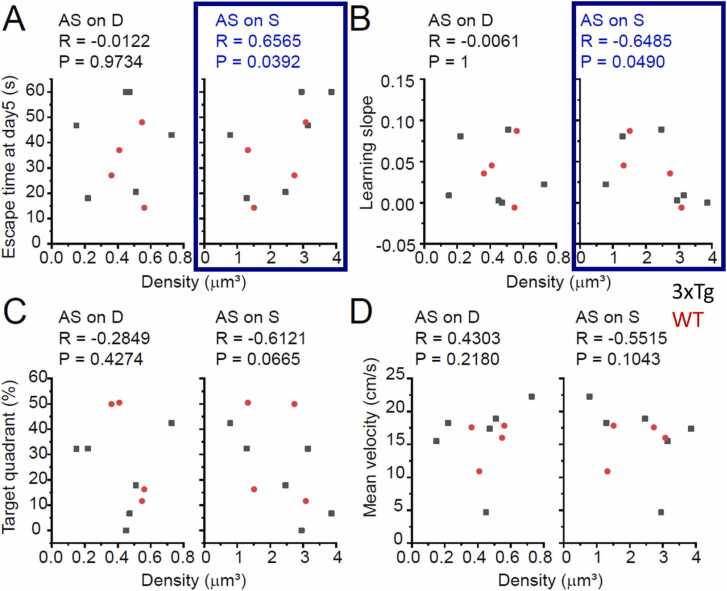
Scatter plot depicting the correlation between the MWM performance and the density of asymmetric, putatively excitatory synapses. **A,B,C**, Relation between the synapse density and MWM performance indicators. The escape latency on Day 5 (A; the shorter the better in learning), the slope of day-by-day shortening of the escape latency (B; the larger the better), and the time spent in the target quadrant (C; the longer the better), are plotted against the density of asymmetric non-spinous synapses on dendrites and somata (AS on D; E-to-N synapses) or on spines (AS on S; E-to-E synapses). Black and red dots represent data from WT and 3xTg mice, respectively. At the top of each diagram, the correlation coefficient (R) and significance level (P) are indicated for the two WT/AD groups combined. In two diagrams enclosed in blue rectangles (A, Right; B, Right), the P value is less than 0.05, indicating significant inverse correlations between the density of asymmetric E-to-E synapses on spines and MWM performance. Shorter escape latencies (A, Right) and larger slopes of learning curves (B, Right) signify the better performances. **D,** The mean velocity is plotted for the control purpose. AS, asymmetric; on D, on dendrites and somata; on S, on spines.

**Fig. 5:**
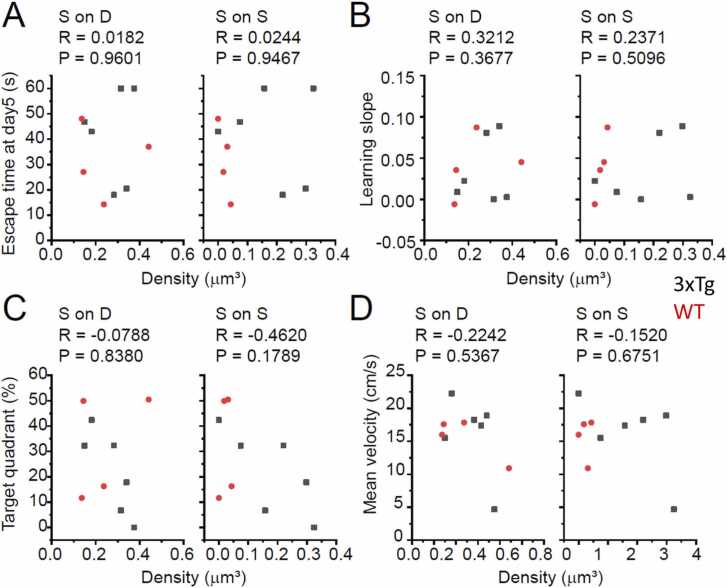
Scatter plot illustrating the correlation of the MWM performance and the density of symmetric, putatively inhibitory synapses. **A,B,C**, Relation between the synapse density and MWM performance indicators. As with Fig. 4, the escape latency on Day 5 (A), the slope of day-by-day shortening of the escape latency (B), and the time spent in the target quadrant (C) are plotted against the density of symmetric non-spinous synapses on dendrites and somata (S on D; I-to-N synapses) or on spines (S on S; I-to-E synapses). Black and red dots represent data from WT and 3xTg mice, respectively. The numerals at the top of each diagram represent the correlation coefficients (R) and significance level (P) for the two WT/AD groups combined. In all the diagrams in this Figure, the P value is more than 0.05, indicating no significant correlations between the density of symmetric synapses and MWM performance. **D**, the mean velocity for the control purpose. S, symmetric; on D, on dendrites and somata; on S, on spines.

The densities of the 4 types of synapses were counted as mentioned. The MWM data were then scatter-plotted against the density of each synapse type, with WT and 3xTg data combined (Fig. 4, Fig. 5). Except for one synapse type, no conceivable correlations were detected between the MWM score and the density. The exception was the density of asymmetric synapses on spines (AsOnSp; E-to-E synapses on spines), which was correlated with the escape latency on day 5 (Fig. 4A right, blue rectangle; R= 0.6565, P = 0.0392), inversely with the slope of the learning curve over the 5 days (Fig. 4B right, blue rectangles; R=-0.6485; P = 0.0490), and therefore pointing to an inverse correlation with spatial learning performance. This is not attributable to the dependence of general motor performance on the density of E-to-E synapses on spines, since the mean swimming velocity is neither correlated with the density of any type of synapses (Fig. 4D, Fig. 5D) nor different between the two genotypes (WT, 16.04 ± 1.53 cm/s, N = 4; 3xTg, 15.38 ± 3.43 cm/s, N = 6; P = 0.8861).

Next, with the AD and WT groups separated, the correlation was examined. In AD mice alone, the Day-5 escape latency or the density of E-to-E synapses was not significantly correlated, though only a vague tendency was suggested (R=0.725, P = 0.103, N = 6). In WT mice alone, the two parameters exhibited no positive correlation (R=-0.312, P = 0.688, N = 4). However, when E-to-E and E-to-N synapses are combined as the whole population of asymmetric synapses, the density was sharply correlated with the Day-5 latency not only in the WT/AD groups combined (R=0.699, P = 0.024, N = 10), but also in AD mice alone (R=0.812, P = 0.04986, N = 6). Therefore, a significant correlation between synaptic excitability and learning performance in AD mice was confirmed in the conventional 2-type classification into asymmetric and symmetric synapses, though such correlation failed to be revealed in the paradigm of our original 4-class synapse categorization.

Finally, in an attempt to clarify the significance of increase in the I-to-E synapse density, correlation was examined between the Day-5 escape latency and the density of this synapse class in AD and WT groups separately. In AD mice, we found the tendency towards a strong correlation (R=0.913, P = 0.058) albeit narrowly outside the significance range, which was not at all observed in WT mice (R=0.319, P = 0.681). A possibility thus arose that the marked increase of I-to-E synapses in AD mice might also contribute to cognitive incompetence.

## Discussion

Hyperexcitability is a prominent feature of cortical network both in AD mouse models and human AD patients, though synaptic loss is also recognized as a hallmark of neuronal degeneration in AD. How synaptic loss compromises hyperexcitability appears to depend on whether it is excitatory or inhibitory synapses that are more severely devastated by AD-associated neurodegeneration. To approach this question, we set out to distinguish asymmetric and symmetric synapses with electron-microscopy, as representative of excitatory (E) and inhibitory (I) synapses, respectively. Then, whether synapses terminate onto spines was adopted as a likely indicator of synapses to excitatory postsynaptic neurons, though those ending onto non-spinous structures (N) cannot be judged to terminate on excitatory or inhibitory postsynaptic neurons. In this way, from hippocampal synapses that we observed, E-to-E and I-to-E synapses are singled out clearly as synapses terminating onto excitatory neurons, whereas the nature of E-to-N and I-to-N synapses was left undetermined. The result showed that (1) the density of hippocampal E-to-E synapses on spines is inversely correlated with MWM performance in WT/AD mice combined, that (2) so is that of the asymmetric synapse consisting of E-to-E and E-to-N synapses in AD mice isolated, and that (3) I-to-E synapses on spines, which are very rare in WT mice, are increased in density in AD mice. This model mouse of 5–6 months old bears no Aβ deposits, is therefore adequate for investigating the toxicity of intracellular Aβ, and has relevance to our previous experiments, in which we used this age range to investigate the mechanism and behavioral consequences of intracellular-Aβ-mediated potassium channel suppression and its reversal (Yamamoto et al., 2011; Wang et al., 2015, Wang et al., 2015).

Brain atrophy and synapse loss are widely recognized as a feature of late-stage AD, just as exhibited in postmortem human AD brains (Perrin et al., 2009, Jack, 2022). In 3xTg mice, by contrast, the absence of neuronal loss in CA1 is reported at 11 months of age (Schaeffer et al., 2017), though the synaptic loss could occur without neuron loss. Baglietto-Vargas et al. (2015) stereologically showed a clear tendency that the number of spines in CA3 pyramidal cells is reduced in 5–6-month-old 3xTg mice as compared to the non-transgenic wild-type, the same age range that we examined, suggesting that synapse loss may take place at E-to-E and/or I-to-E synapses onto spines. Our present observation of increase in I-to-E synapses on spines, combined together with the spine loss that Baglietto-Vargas et al. (2015) reported, suggest that E-to-E synapses and I-to-E synapses on spines are decreased and increased, respectively, during the progression of AD pathology in 3xTg mice. Such synaptic adjustment may represent rearrangement of spinous inputs to counteract excitotoxicity and neuron loss, which would agree with the lack of neuron loss in 11-month-old 3xTg mice (Schaeffer et al., 2017). At the very least, the present study showed that some synapses are proliferated in AD model brains, rather than lost, in a local landscape within the big picture that unfolds a monotonous loss of neural tissue as the hallmark of AD progression (Perrin et al., 2009, Jack, 2022).

Synapse loss in the hippocampus has been investigated in diverse mouse models of AD. Decreased numbers of dendritic spines were reported in J20 mice (Palop et al., 2007, Mairet-Coello et al., 2013), APP/PS1 mice (Wang et al., 2023), and 5xFDA mice (Herzer et al., 2018), which suggested in a surrogate way a synaptic loss without specifying the excitatory and inhibitory nature of presynaptic terminals. In 3xTg mice, Baglietto-Vargas (2015) reported that the total spine density in the hippocampus is reduced as compared to WT mice, whereas that of the mushroom-shaped spine, which is the most matured and conspicuous among all the types of spines including the thin and stubby types, did not differ between the two genotypes. This finding was confirmed both in 3xTg mice and the APP^NL-G-F^ type of knock-in mice (Carpanini et al., 2022). Since the thin and stubby types are considered to be immature and under development to the matured mushroom type that are much more voluminous and fully engages in synaptic transmission, the cross-section of these two immature types may be underrepresented in morphological studies as compared to the mushroom type. Cross-sections of mushroom spines would then be overrepresented and have a higher probability to be observed and analyzed, and it could be such bias that may have obscured a potential difference in the E-to-E synapse density in the present study. Furthermore, Mijalkov et al. (2021) reported that dendritic spines on hippocampal pyramidal cells are lost not homogenously but in cluster in AD patients’ brains, which suggests that where specimen for EM is sampled from might critically affect the result. If this held true to 3xTg mice, our sampling would not necessarily be unbiased.

As for symmetric I-to-E synapses, which are much fewer than asymmetric E-to-E synapses on spines, the analysis on spines alone could not, in principle, address this synapse type directly. An EM study in APP/PS1 mice (Keller et al., 2025) reported that the density of asymmetric synapses on spines, which are most likely excitatory synapses on excitatory neurons, was reduced in the hippocampus, while much fewer symmetric synapses (2–5 % over total synapses) were not analyzed in details. This type of synapses has been scarcely dealt with because of their paucity, and therefore no clear account appears to be available to date. In this situation, our study suggested a marked tendency for the I-to-E synapse density to corelate with the Day-5 escape latency in AD mice, raising the possibility that, along with E-to-E synapses, the increased I-to-E synapses in AD mice might also contribute to cognitive incompetence represented by elongation of the escape latency, even though it would rather curtail the hyperexcitability characteristic to AD mice. This might sound contradictory, given the known view that the hyperexcitability observed widely in AD mice is harmful to cognition (Palop et al., 2007, Busche et al., 2012; Wang et al., 2015, Wang et al., 2015; Zott et al., 2018, Zott et al., 2019). However, since E-to-E synapses are much denser than I-to-E synapses, the impact of I-to-E synapses on the overall excitability may be negligible. Moreover, our study is based on a very small number of counts, and therefore errors attributable to sampling bias could not be completely ruled out in the present assessment of I-to-E synapses. For the same reason, the increase in this type of synapses that we found would have much less impact on the overall density of hippocampal synapses or spines. Also, one must be cautioned about the small sample number of mice here.

The present study demonstrated an inverse correlation between the density of hippocampal E-to-E synapses on spines and the MWM performance, thereby pointing to a deleterious effect of hyperexcitability on cognition. Although the correlation was significant in the AD/WT groups combined, only a narrowly non-significant tendency (R=0.913, P = 0.058) was observed in the AD group isolated. Interestingly, however, once we leave our original 4-class synapse categorization and take E-to-E and E-to-N synapses together as the asymmetric synapse class, the correlation turned out to be significant in AD mice under the conventional 2-type classification into asymmetric and symmetric synapses that pays no attention to the postsynaptic nature, and clearly supports the well-known view that hyperexcitability harms cognition in AD mice (Born, 2015; Zott et al., 2019).

It is yet to be determined whether the hyperexcitability in AD mice affects the markedly increased density of I-to-E synapses in a compensatory manner. Our previous experiments by RT-PCR demonstrated that expression of the activity-dependently inducible immediate early gene Homer1a is higher in the neocortex of 3xTg than WT mice, agreeing with an elevated excitability in 3xTg mouse neurons (Wang et al., 2015, Wang et al., 2015). We also demonstrated that intracellular Homer1a activates BK-type potassium channels, thereby reducing excitability in a negative feedback fashion in AD as well as WT mice (Yamamoto et al., 2011). Irrespective of whether compensation for hyperexcitability takes place or not, the combination of the hyperexcitability and increased I-to-E synapses would result in a disruption of cortical excitatory-inhibitory balance during the early phase of AD pathogenesis, which may also contribute to devastating cognition. Conceptually, such imbalance may represent a shift of homeostatic balance between synaptic transmission and intrinsic excitability within single neurons, which may have shifting equilibrium points adopted differentially to developing, mature, and degenerating brains. In AD brains, intrinsic hyperexcitability may be partly alleviated by activity-dependent homeostatic plasticity (Turrigiano and Nelson, 2004) that fosters inhibitory transmission.

Lines of research have elucidated mechanisms for AD-accompanied hyperexcitability. Our previous experiments attributed Aβ-induced hyperexcitability of 3xTg neocortical pyramidal neurons to intracellular-Aβ-induced blockade of big-conductance calcium-activated potassium (BK) channels (Yamamoto et al., 2011, Wang et al., 2015, Wang et al., 2015). We also showed Aβ accumulation in amygdala principal neurons in 3xTg mice, which exhibited hyperexcitability caused by intracellular Aβ as well (Xu et al., 2021). Involvement of other types of K channels in Aβ-induced neuronal excitability has also been reported; the delayed rectifier, transient outward, and small-conductance calcium-activated K (SK) channels (Alier et al., 2011, Scala et al., 2015). Besides K channels, neurons that were derived from iPS cells collected from AD patients displayed increased sodium current density and increased excitatory and decreased inhibitory synaptic activities in agreement with the hyperexcitable nature of AD patients’ nervous system (Ghatak et al., 2019). AD-related hyperexcitability thus induced by various mechanisms may well underpin cognitive deterioration in AD patients or models, and also promote the proposed positive feedback production of Aβ by hyperexcitability (Cirrito et al., 2005, Yamamoto et al., 2011, Scala et al., 2015), which would further impair cognition.

## CRediT authorship contribution statement

**Tetsufumi Ito:** Writing – original draft, Methodology, Investigation, Funding acquisition, Formal analysis, Data curation, Conceptualization. **Munenori Ono:** Writing – original draft, Methodology, Investigation, Funding acquisition, Formal analysis, Data curation, Conceptualization. **Sachiko Yamaki:** Investigation, Funding acquisition. **Yoshie Hori:** Investigation. **Shinji Muramoto:** Investigation. **Ryo Yamamoto:** Investigation, Funding acquisition. **Takafumi Furuyama:** Investigation, Funding acquisition. **Nobuo Kato:** Writing – review & editing, Writing – original draft, Validation, Supervision, Project administration, Investigation, Funding acquisition, Formal analysis, Data curation, Conceptualization.

## Funding

This work was supported by the 10.13039/501100001691Japan Society for the Promotion of Science through KAKENHI Grants JP16K11200 (MO), 23H04342 (TI), 24K10746 (RY), 21K11251 (SY), 22K15795 (TF), 17H02223 (NK), 22K09734 (NK) by the JST FOREST Program JPMJFR2151 (TI), and by Grants from Smoking Research Foundation (TI), Shibuya Science Culture and Sports Foundation (NK, MO), the Naito Foundation (TF), and Kanazawa Medical University: S2016–8 (MO) C2018–1 (NK) C2022–3 (RY).

## Declaration of Competing Interest

The authors declare that they have no known competing financial interests or personal relationships that could have appeared to influence the work reported in this paper.

## Data Availability

Data are available from the corresponding author upon reasonable request.
